# The Neuroimmunology of Guillain-Barré Syndrome and the Potential Role of an Aging Immune System

**DOI:** 10.3389/fnagi.2020.613628

**Published:** 2021-01-13

**Authors:** Kathleen M. Hagen, Shalina S. Ousman

**Affiliations:** ^1^Department of Neuroscience, Hotchkiss Brain Institute, University of Calgary, Calgary, AB, Canada; ^2^Departments of Clinical Neurosciences and Cell Biology and Anatomy, Hotchkiss Brain Institute, University of Calgary, Calgary, AB, Canada

**Keywords:** peripheral nervous system, PNS, guillain-barré syndrome, GBS, immune system, aging, gangliosides

## Abstract

Guillain-Barré syndrome (GBS) is a paralyzing autoimmune condition affecting the peripheral nervous system (PNS). Within GBS there are several variants affecting different aspects of the peripheral nerve. In general, there appears to be a role for T cells, macrophages, B cells, and complement in initiating and perpetuating attacks on gangliosides of Schwann cells and axons. Of note, GBS has an increased prevalence and severity with increasing age. In addition, there are alterations in immune cell functioning that may play a role in differences in GBS with age alongside general age-related declines in reparative processes (e.g., delayed de-differentiation of Schwann cells and decline in phagocytic ability of macrophages). The present review will explore the immune response in GBS as well as in animal models of several variants of the disorder. In addition, the potential involvement of an aging immune system in contributing to the increased prevalence and severity of GBS with age will be theorized.

## Overview of the Immunology of the Peripheral Nervous System

In the intact peripheral nerve, there are many cell types that reside. Among them are neurons, Schwann cells (SCs), fibroblasts, pericytes, endothelial cells, and resident macrophages. After sufficient injury to peripheral nerves, other immune cells such as infiltrating macrophages, neutrophils, mast cells, dendritic cells, and T cells contribute to degenerative and regenerative processes. For example, myelinating and non-myelinating SCs de-differentiate into a repair phenotype that secretes cytokines, chemokines, and neurotrophic factors. In addition, several beneficial processes occur: bands of Büngner are formed by repair SCs; neutrophils and macrophages are recruited for debris clearance alongside repair SCs (Gaudet et al., 2011); and activation of mast cells increase blood-nerve barrier permeability (Olsson, 1967; Esposito et al., 2002). Other responses are also noted including recruitment of various pro- and anti-inflammatory T cell types (Xin et al., 2008; Austin et al., 2012), modulation of SC sorting and cytokine production by fibroblasts (Reichert et al., 1996; Parrinello et al., 2010), and the secretion of neurotrophic factors and axon guidance molecules by endothelial cells (Cattin et al., 2015; Grasman and Kaplan, 2017).

Of note, there is augmented severity and/or prevalence of immune cells within the PNS with increasing age. For instance, there is an increased presence of mast cells and macrophages not only in the uninjured nerve (Ceballos et al., 1999), but also after injury (Büttner et al., 2018; Stratton et al., 2020). As reviewed by Verdú et al. (2000), this heightened immune response may contribute to the decreased regenerative ability of nerves with age. That is, it does not appear to be an inability of the axons themselves to regrow with increasing age, but rather it appears to be deficits in the aged nerve environment that are involved (Kang and Lichtman, 2013). For example, there is an age-related decline in SC de-differentiation that may delay the clearance of debris following degeneration and thus impair the growth of regenerating axons (Painter et al., 2014). In addition, with age, there is a delay in macrophage recruitment and a defect in the ability of macrophages and SCs to phagocytose debris (Scheib and Höke, 2015).

Since injury-related immunological responses in the injured PNS have been reviewed in detail in the past (e.g., Gaudet et al., 2011; DeFrancesco-Lisowitz et al., 2014; Mietto et al., 2015), we will focus here on the immunological aspects of GBS, a disorder that involves non-mechanical damage of peripheral nerves, by discussing studies involving the immune system in the disease. This will be accompanied by the authors' views on age-mediated changes in the immune system in addition to the aforementioned alterations to macrophages and SCs that could account for the particular severity of the disease seen in older GBS humans.

## Guillain-barré Syndrome (GBS)

### Incidence Rate

Guillain-Barré syndrome (GBS) has a general incidence rate of between 1.1 and 1.8/100,000 per year (McGrogan et al., 2009). It typically affects more men than women in a 1.25:1 ratio (Hughes and Rees, 1997). When broken down by age in a meta-analysis study that examined incidence rates from North American and European epidemiological reports, the prevalence of GBS was found to change dramatically (Sejvar et al., 2011). In those aged 0–9-years-old, the incidence rate was found to be 0.62/100,000 per year, specifically 0.8 in males and 0.45 in females. However, the rate increases by 20% per decade of life leading to a prevalence of 2.66/100,000 per year in those aged 80–89 years (specifically 3.72 in males and 2.09 in females). It should be noted that older studies examining the prevalence of GBS have reported that there is a decrease in the incidence rate over the age of 75 years of age (George and Twomey, 1985); this difference in findings could be due to improvements in diagnostic methods. Of further note and pertinent to the subject of this review, Hughes and Rees speculated early on that the higher incidence rate in older populations may reflect a failure in the typical suppression of the immune system with increasing age (Hughes and Rees, 1997).

### Severity

In addition to higher incidence rates of GBS in the elderly, one study found greater severity of the disorder at its peak as well as a higher mortality rate in older individuals compared to younger patients (Peric et al., 2016). In the aforementioned study, the authors striated their patients into young (<60-years-old), young-old (60–80-years-old), and old-old (>80-years-old). While the severity of disease at peak was not significantly different between young-old and old-old patients, there was more disability at discharge, more frequent involvement of bulbar symptoms, and higher rates of comorbidity in the old-old group compared to the young-old patients. Furthermore, in a study of clinical predictors of plasmapheresis treatment, age was the only predictor, with young patients responding better than older patients (Gruener et al., 1987).

### Symptoms and Fluid Bio-factors

GBS is broadly characterized by areflexia and symmetrical limb weakness which typically begins proximally and can involve motor, sensory, and/or cranial nerves (George and Twomey, 1985; van Doorn et al., 2008). GBS symptoms usually progress, typically lasting around 4 weeks from onset (Ho et al., 1997) and can present with autonomic dysfunction that can lead to changes in mental state (Lehmann et al., 2010), fatigue, and pain (van Doorn et al., 2008). The disease has also been associated with increases in cerebrospinal fluid (CSF) levels of protein, albumin (Li et al., 2012), neuroactive steroids such as dehydroepiandrosterone sulfate (Azuma et al., 1993), malondialdehyde and antioxidant activity (Ghabaee et al., 2010), haptoglobin, α-1-antitrypsin, apolipoprotein A-IV, and neurofilament (Yang et al., 2008), and complement products C3a and C5a (Hartung et al., 1987). On the other hand, decreases in Cystatin C, transthyretin, apolipoprotein E, and heat shock protein 70 (Yang et al., 2008) in the CSF have been noted.

Molecular alterations are also observed in the serum of GBS patients. For instance, Ghabaee et al. found decreases in serum malondialdehyde and antioxidant activity (Ghabaee et al., 2010). Other groups have also examined lipophilic antioxidants in the context of GBS, however they did not find a significant difference in the concentrations of malondialdehyde in patient plasma as in the previously mentioned study, nor did they find a difference in myeloperoxidase concentrations (Tang et al., 2017a). However, they did find a significant decrease in the concentrations of γ-tocopherol and δ-tocopherol and an increase in α-carotene concentrations in GBS patients compared to controls. The latter observation may be indicative of increased risk to oxidative stress-induced damage. In addition, it was noted that GBS patients had a lower concentration of cholesterol compared to controls, although this may reflect demyelination. In a metabolomics analysis in GBS patients, it was found that patient plasma had lower levels of aspartate, creatinine, serotonin, taurine, phosphatidylcholines, lysophosphatidylcholines, sphingomyelins, and acylcarnitines, and higher levels of isoleucine compared to controls (Tang et al., 2017b). Thus, there are alterations in a wide variety of metabolic factors and the question is whether any are the primary mediators of disease or simply markers of damage.

### Etiology

GBS has been associated with preceding infections in approximately two-thirds of patients (Hughes and Rees, 1997); these infections typically involve the digestive or respiratory systems and are the basis for an underlying “molecular mimicry” causation (reviewed in Jasti et al., 2016). Some examples of common infections associated with GBS include *Cytomegaloviru*s, *Epstein–Barr virus, Herpes, Hepatitis A and E, Human Immunodeficiency Virus, Hemophilus influenza, Mycoplasma pneumoniae*, and *Campylobacter jejuni* (Dalakas, 2015).

### The Immunology of GBS

First identified in 1969 by Asbury and colleagues, a common characteristic of many GBS patients is a prominent lymphocyte infiltration into the nerves (Asbury et al., 1969). The authors also found that consistent inflammation was seen even in patients who had recovered and therefore speculated that this could be the underlying basis of a relapse. Since then, significant work has gone into identifying what immune cell populations are involved in the inflammation. For example, T cells have been identified in the endoneurium and the epineurial space near venules in sural nerve biopsies of GBS patients, and of these infiltrating T cells, both CD4^+^ and CD8^+^ phenotypes were seen (Schmidt et al., 1996). Along with T cells, there is an increase in macrophages in the endoneurium and epineurium of these nerves. Further, Yoshii and Shinohara noted that natural killer cell activity was found to be decreased in GBS patients compared to controls; the authors speculated that deficits in natural killer cell function could leave individuals at a predisposition to acquire GBS because of the immune suppressing ability of these cells (Yoshii and Shinohara, 1998). The majority of work in GBS research has however, focused on T cells. Interestingly, it is known that clonal expansion occurs when T cells are activated, and when this expansion happens, it can lead to random mutations in their genome. It has been shown that there is a higher frequency of hypoxanthine-guanine phosphoribosyltransferase mutant T cells in patients with GBS compared to healthy controls with the frequency of the mutations lessening during the recovery phase (Van den Berg et al., 1995).

#### Gamma Delta (γδ) T Cells

Of the various types of T cells, Borsellino and colleagues concluded in a review of the literature, that the Vδ1 subset of γδ T cells was the most prevalent in GBS as it has been found to be at three times its normal numbers in patients (Borsellino et al., 2000). Also, in some patients that had become infected with *C. jejuni*, there is an enrichment in the γδ T cell population, specifically of the Vγ5/Vδ1 subset (Ben-Smith et al., 1997). In addition, γδ T cells have been seen in nerve biopsies of acute inflammatory demyelinating polyradiculoneuropathy (AIDP) patients, a variant of GBS (Khalili-Shirazi et al., 1998). γδ T cells have the ability to recognize non-protein epitopes which means they may be able to recognize carbohydrates in gangliosides and ganglioside complexes and contribute to the immune response against *C. jejuni*, thus promoting molecular mimicry (Winer et al., 2002).

#### Aging γδ T Cells and GBS

With age in the typical immune system, the number of γδ T cells decreases which contrasts with what is seen in GBS where there is an increase in the number of γδ T cells; however, the age-related reduction in γδ T cells has been associated with the Vδ2 subset rather than the Vδ1 subset (Michishita et al., 2011). Furthermore, when the total number of γδ T cells and the Vδ2 subset were quantified in age-matched pairs, it was discovered that although there were significantly higher numbers of these populations in males than in females, no differences in the Vδ1 subset were evident. Interestingly, when age was considered both males and females had a significant negative correlation in Vδ2 T cell numbers (i.e., *R* = −0.462 in males, *R* = −0.330 in females). Thus, alteration in the number of Vδ1 cells in GBS may not be a major contributor to possible age-related immunological drivers of the disease.

However, in terms of the activation profile of γδ T cells during aging, De Rosa et al. found that within 1 year of life, the majority of γδ T cells, and specifically Vδ2 T cells, showed signs of previous activation (i.e., memory T cells) (De Rosa et al., 2004). The authors hypothesized that a portion of γδ T cells may be responsive to self antigens because after an examination of the umbilical cord blood of newborn infants, they found that a portion of both Vδ1 and Vδ2 T cells were non-naïve which could only have occurred prior to exposure to external antigens. This is possible since γδ T cell populations are found in barrier tissues throughout the body (Khairallah et al., 2018). Also, because of their location and exposure to antigens, they appear to be able to form a memory population. In the context of GBS, the question is whether the observed increase in γδ T cells (Ben-Smith et al., 1997; Khalili-Shirazi et al., 1998) is due to tissue-resident γδ T cells responding to gangliosides that resemble previously encountered antigens. Some evidence to support this possibility was shown by Spada et al. who found that Vδ1 γδ T cells have the ability to recognize and attack CD1c^+^ antigen presenting cells (APCs) (Spada et al., 2000). Further, the γδ T cells that were reactive to CD1c^+^ APCs produced Th1-like cytokines. The authors concluded that this was a method for the immune system to target these cells in the absence of foreign antigen via both perforin- and Fas-dependent cytotoxicity. It is plausible that the increase in γδ T cells seen in GBS may be an attempt to regulate the immune response thereby removing CD1c^+^ APCs (e.g., B cells, activated macrophages). Indeed, there is data that this may be occurring in humans. For instance, Vasudev et al. showed in elderly individuals, there was a decrease in the percentage of CD8^+^ and γδ T cells compared to the young, while there was an increased percentage of CD4^+^ T cells in the elderly compared to the young (Vasudev et al., 2014). It has also been found that γδ T cells from old (66–96 years of age) and very old (99–103 years of age) individuals are pre-primed in that they have an increased expression of CD69 and increased basal TNF-α secretion compared to young individuals (20–48 years of age) (Colonna-Romano et al., 2002). However, upon stimulation, these aged subjects' cells produced less TNF-α compared to the young. Furthermore, those over 100 years of age had an increased percentage of apoptotic γδ T cells compared to old (aged 75–94) and young (aged 24–53) individuals (Colonna-Romano et al., 2004). Because of the proneness of these cells to undergo apoptosis with increasing age, perhaps there is less of a “brake” on the immune response to preceding infections, a speculation that would favor the molecular mimicry hypothesis that is linked to GBS.

#### CD4^+^ CD25^+^ T Cells

In a study by Pritchard et al., it was found that there was a reduction in the number of CD4^+^ CD25^+^ T cells in GBS patients in the first 1–2 weeks of the disease compared with healthy controls; no differences were seen in the numbers of total B cells, CD5^+^ B cells, memory B cells, γδ T cells, natural killer cells, or CD16^+^ natural killer T cells (Pritchard et al., 2007). There were also less CD4^+^ CD25^+^ T cells expressing HLA-DP, DQ, DR in the GBS patients compared to healthy controls. Among the patients with the lowest numbers of CD4^+^ CD25^+^ T cells, 4 of the 6 patients had antibodies against gangliosides while only 3 out of 14 patients with slightly higher numbers of CD4^+^ CD25^+^ T cells tested positive for those antibodies. It is worth noting that after the first 1–2 weeks of the disease, the majority of patients in the study had received intravenous immunoglobulin (IVIg) therapy which may have contributed to the return of CD4^+^ CD25^+^ T cell numbers to control levels. In keeping with these findings, Chi et al. also reported reductions in CD4^+^ CD25^+^ T cells in the initial stages of GBS and an increase in their numbers following IVIg therapy (Chi et al., 2007). The authors also examined the functionality of these T cells between patients and controls and found that both groups' cells showed equivalent expression of FoxP3 mRNA and suppressor functions (i.e., reduced proliferation and cytokine secretion in responder cells) when co-cultured with CD4^+^ CD25^+^ effector T cells. From this, the authors concluded that the initiation of GBS could be due to a short-term reduction of this CD4^+^ CD25^+^ regulatory T cell population and the reversibility of this effect may contribute to the monophasic course of the disease. Harness and McCombe also examined T cell populations in GBS peripheral blood mononuclear cells (PBMCs) and compared them with other neuropathies and healthy controls (Harness and McCombe, 2008). In the GBS patients, they found a reduction in the percentage of CD4^+^ CD25^+^, CD3^+^, and CD8^+^ T cells and total cell population expression of CD95 (Fas), while an increase in Bcl-2 expression within the CD4^+^ CD25^+^ T cell population was noted. The reduced expression of CD3^+^ T cells was explained by a reduction in CD8^+^ T cells.

#### Aging Regulatory T Cells and GBS

In terms of age and regulatory T cells (Tregs), Hwang et al. have examined the frequency of regulatory T cells in peripheral blood and found that there was no significant difference between young and old individuals; however within regulatory T cells from older individuals there was an increase in non-naïve regulatory T cells compared to young individuals (Hwang et al., 2009). In addition, their main functional change with age appeared to lie in their ability to suppress interleukin (IL)-10. That is, in an experiment comparing co-cultures of regulatory T cells with CD4^+^ effector cells, the aged cells (samples from patients >65 years of age) were better at suppressing IL-10 production compared to T cells from younger (<40 years of age). However, the aforementioned effect was only seen when T cells were cultured in a one-to-one ratio, which would not be the case in the context of GBS in which there is a decrease in regulatory T cells.

While Hwang et al. reported no difference in frequency of regulatory T cells between ages in peripheral blood in humans, others have reported that there is actually an increase in regulatory T cells in aged individuals (Gregg et al., 2005; Rosenkranz et al., 2007; Raynor et al., 2012). This increase in Tregs appears to be caused by a down-regulation of Bim which is an apoptotic factor (Chougnet et al., 2011). There are no studies to date that have assessed the number of regulatory T cells or the function of these cells in GBS in the context of aging. Thus, it is unknown whether there is an increase in regulatory T cells like that seen with normal aging. One can speculate that even if there was an increase, it would be below that of normal aging because of the fewer numbers of these cells in GBS individuals. Thus, one can further hypothesize that fewer regulatory cells in elderly people could contribute to the increased severity of the disease in this population.

#### T Cell Cytokines

##### T Helper (Th)1 and Th2

When analyzing GBS patient lymphocytes stimulated with *C. jejuni*, it was found that in the initial stages of the disease there is an upregulation of Th1 pro-inflammatory cytokine mRNA [i.e., interferon-γ (IFN-γ), IL-1β, tumor necrosis factor (TNF), IL-6, IL-10, and IFN-γ:IL-4 ratio] and a decrease in Th2 anti-inflammatory cytokine mRNA (i.e., transforming growth factor (TGF)-β and IL-4) (Nyati et al., 2011). However, in the recovery phase, there is a predominant upregulation of Th2 cytokines and a decrease in the expression of Th1 cytokines. IFN-γ is not the only Th1 cytokine dysregulated in people with GBS. GBS PBMCs produce less IL-2 and have a lessened response to IL-2 compared to healthy controls, however this effect dissipated after treatment with plasma exchange (Yoshii and Shinohara, 2000). There was also an increase in soluble IL-2 receptors which decreased over the disease course. The authors postulated that this could be due to a functional depression in T cells due to a previous reaction to a preceding infection and therefore may be allowing the body to recover from the previous immune response.

##### Th17

There is a growing body of evidence supporting the role of Th17 cells and their pro-inflammatory cytokines in the pathology of GBS (Wu et al., 2016). The levels of IL-17 and IL-22 are elevated in the plasma and CSF of patients with GBS compared to healthy controls, and further, the CSF levels of the two cytokines correlate with disease score severity (Li et al., 2012). It is speculated that the presence of the cytokines in CSF may reflect a disruption in the blood-nerve barrier (BNB). In terms of mechanisms, Liang et al. isolated PBMCs from GBS patients and controls and examined mRNA expression of T cell immunoglobulin and mucin-3 (TIM-3), which is highly expressed on Th1 cells and is thought to decrease IFN-γ secretion when bound (Liang et al., 2012). They also examined the presence of Th17 cells. In CD4^+^ T cells, the group found a decrease in the expression TIM-3 and an increase in the expression of RORγt. When anti-TIM-3 antibodies were given, secretion of IFN-γ and IL-17 increased in GBS compared to controls. Further, there was an increase in the number of Th17 cells compared to Th1 and Th2 cells in the circulation of GBS patients. The authors concluded from these findings that TIM-3 may play an inhibitory role in Th17 activation. In alignment with this study, other groups have also reported increased levels of Th17 cells, increased mRNA expression of RORγt, and increased IL-17 concentrations in the plasma and CSF of GBS patients (Han et al., 2014). For example, Li et al. studied the prevalence of Th1, Th17, and Th22 cells in GBS compared to controls and other conditions and found that GBS patients had increased frequency of Th1, Th17, and Th22 cells in their circulation along with elevated IL-17 and IL-22 in their serum (Li et al., 2014). Of further note, IVIg therapy led to a decrease in serum levels of IL-17 and IL-22 and a general decrease in Th17 and Th22 cells.

#### Aging T Helper Cells, Their Cytokines, and GBS

Because of thymic involution and the decline in peripheral naïve T cell proliferation with age, there is a significant disruption in the production of naïve T cells as one grows older (Haynes et al., 2000; den Braber et al., 2012). This would impact Tregs and Th17 cells that are both derived from naïve T cells (Lynch et al., 2009). During typical aging there is an increase in Tregs, however in GBS patients there is a decrease in Tregs and an increase in Th17. Perhaps because there are so few naïve T cells with age, there is an imbalance in the differentiation of Th17 and Tregs once a concurrent infection triggers the immune response via γδ T cells in GBS patients. Indeed, Schmitt et al. have shown that the balance between Th17 cells and Tregs changes with age (Schmitt et al., 2013). Specifically, they noted that in unstimulated cells, there was an increase in the Th17/Treg ratio with increasing age, but when stimulated, there was a decrease in the Th17/Treg ratio with increasing age. Interestingly, Lim et al. have shown that in aged mouse splenocytes, there is an increased tendency to produce Th17 cells compared to cells from young mice (Lim et al., 2014). In addition, increased IL-1β and decreased IL-2 signaling associated with age are important factors in the upregulation of Th17 cells.

IL-2 is another cytokine that is important for the expansion of regulatory T cells (Nelson, 2004). As previously mentioned, Yoshii and Shinohara had demonstrated that PBMCs from GBS patients produce less IL-2 and have a lessened response to IL-2 compared to healthy controls (Yoshii and Shinohara, 2000). In addition, with age, there is reduced production and response to IL-2 (Rabinowich et al., 1985; Pahlavani and Richardson, 1996). Altogether, this could indicate a deficiency in IL-2 impairing normal age-related accumulation of regulatory T cells and thus leading to an increase in the susceptibility of acquiring GBS with age.

In addition to a decline in naïve T cells via thymic involution and decreases in peripheral T cell proliferation, naïve T cells from older individuals produce less IL-2 which would negatively affect the generation of effector T cells (Haynes et al., 2000; den Braber et al., 2012). When IL-2 was exogenously given, it rescued effector cell expansion and defective functioning in aged naïve T cells. However, while in the effector state, aged CD4^+^ T cells can produce equivalent IL-2 levels to young CD4^+^ T cells when exposed to exogenous IL-2. Interestingly, when these exogenously IL-2-exposed effector T cells from aged animals convert to memory T cells, they no longer produce equivalent IL-2 levels, instead they revert back to low IL-2 production when re-stimulated. This may be related to the increase in Th1 cytokines and a decrease in Th2 cytokines seen early in the disease course of GBS; this effect is reversed during the recovery phase of GBS (Nyati et al., 2011). Because IL-2 is mainly produced by Th1 cells (Pahlavani and Richardson, 1996) and is needed for effector T cell expansion (Nelson, 2004), this could explain the increase in helper T cells seen in GBS. While there is an increase in helper T cells in GBS, with age there is a decrease in IL-2 production and responsiveness and thereby a decline in Treg expansion as previously mentioned (Rabinowich et al., 1985; Pahlavani and Richardson, 1996), thus allowing helper T cells to function unregulated in the context of GBS.

The cytokines produced by Th1 and Th17 cells could lead to defects in APCs such as macrophages. There are more macrophages present in the intact nerves of older mice relative to that of a mature adult mouse (Büttner et al., 2018). In addition, there is an upregulation of IL-4 in sciatic nerves from adult animals, however in older animals, this upregulation is no longer seen. IL-4 is needed to induce an anti-inflammatory phenotype in macrophages and with age, macrophage responses to IL-4 are altered (Mahbub et al., 2012; Albright et al., 2016). As GBS progresses and the inflammatory landscape resolves, the upregulation of Th2 cytokines, specifically IL-4, may contribute the recovery of the disease via induction of phenotypic shifts in macrophages from pro-inflammatory to anti-inflammatory.

#### Other Immune-Contributing Factors

##### BNB Permeability

Another potential mechanism underlying nerve damage in GBS could be “bystander” inflammation (Harvey et al., 1995). In one study, T cells sensitized to ovalbumin were transferred into Lewis rats followed by an intraneural injection of ovalbumin or casein. In the ovalbumin-injected group, there was a rapid influx of immune cells into the nerve, specifically αβ T cells and macrophages. The increase in cells into the nerve was consistent with increased BNB permeability which is notable since increased permeability has the added detrimental effect of opening up the nerve environment to circulating neural-specific antibodies (Pollard et al., 1995).

##### CD1 Polymorphisms

Polymorphisms on the CD1 gene in humans have been associated with increased risk of developing GBS. Individuals with CD1E^*^01/01 genotype have an increased likelihood of developing GBS while those with a CD1A^*^01/02, CD1E^*^01/02, or both have a decreased chance in developing GBS (Caporale et al., 2006a).

##### Integrins

In GBS patients, differences in integrin expression have been found relative to controls (Previtali et al., 1998). Specifically, the expression of β4 integrins was absent from both myelinating and non-myelinating SCs, and in sural nerves with axonal loss. SCs were also found to express α5 integrins, but it is unknown what role these integrins play.

#### Antigen(s)

Many studies have sought to identify the target antigen of the immunological response in GBS. Work by Terryberry et al. found a strong correlation between the presence of autoantibodies against tubulin and neurofilament-H in GBS patient serum (Terryberry et al., 1998). Further, Notturno et al. found that 71% of GBS patients possessed antibodies against single gangliosides or ganglioside complexes (Notturno et al., 2009). These results have been strengthened with others finding an association between antibodies against ganglioside complexes and preceding infections. For example, Kaida et al. showed that 17% of the GBS patients in their study had antibodies against a ganglioside complex and that those who had anti-monosialotetrahexosylganglioside (GD)1a/GD1b and/or anti-GD1b/polysialoganglioside (GT)1b antibodies had an increased association with the need for mechanical ventilation (Kaida et al., 2007). Interestingly, patients with GD1a/GD1b and/or anti-GD1b/GT1b antibodies had sera that reacted with GM1/GD1a and GM1/GT1b. Gangliosides serve many purposes, but among them is protection from complement activation via the binding of complement regulatory protein factor H (Cutillo et al., 2020). Given that GBS is often associated with anti-ganglioside antibodies, it could be that these antibodies are allowing for the activation of the complement system by attacking gangliosides and thus leading to further recruitment of immune cells. In addition, anti-GM1 antibodies have been shown *in vitro* to facilitate leakage of the BNB thus allowing for penetration into the nerve environment (Kanda et al., 2000).

Because of the known complement activation see in GBS patients, recent clinical trials have examined the role of eculizumab (a humanized monoclonal antibody against C5 that prevents cleavage into C5a and C5b components) in patients with GBS (Davidson et al., 2017; Misawa et al., 2018). Results of a Japanese, multicenter, Phase 2 clinical trial showed that 14 of 23 patients were able to walk independently at the conclusion of the trial in the eculizumab group compared to 5 of 11 patients in the placebo/IVIg group. However, of the 34 GBS patients enrolled in the study, three experienced serious adverse effects (Misawa et al., 2018). Also, due to the small sample size, statistical comparisons between the eculizumab and placebo groups could not be performed. Earlier examination of eculizumab in humans had been performed in the United Kingdom, however again, sample size was very small due to low recruitment levels (Davidson et al., 2017). In this study, patients were randomly assigned to the IVIg with eculizumab or IVIg with placebo groups. After pre-screening and informed consent of GBS patients, only seven patients were eligible with five being assigned to the eculizumab group and two patients being given placebo. In this trial, four serious adverse events were reported. In terms of primary outcomes, both placebo controls and two of the five eculizumab-receiving patients experienced a decrease in GBS disability score by at least one grade. Taking both studies together, it appears that eculizumab may be effective in severe cases although given the sample sizes of the aforementioned studies, there is need for more studies on the effectiveness and possible adverse consequences of the treatment (Misawa and Suichi, 2020).

#### Gangliosides With Age

The composition of gangliosides within the CNS undergoes dramatic changes with age (Kracun et al., 1991), and although the composition of gangliosides differs between the CNS and PNS, it was hypothesized that perhaps similar age-related alterations in ganglioside composition are seen in the PNS as in the CNS. Ohsawa tested this idea by examining the ganglioside composition in dorsal root ganglia (DRG) of rats across their lifespan up to 24 months of age (Ohsawa, 1990). From birth to 12 months of age, there was an increase in total gangliosides within the DRG, but this coincided with an increase in the weight of the DRG. From 12- to 24-months of age, the total gangliosides continued to increase, but much less rapidly. This latter observation also occurred in parallel with an increase in DRG weight. The question then is whether the increase in ganglioside content is simply related to the increased size of the animal. Importantly also, in terms of composition, Ohsawa found that after the first few postnatal months, there was relatively little alteration in the composition of gangliosides within the DRG. However, it is unknown whether ganglioside content is changed with age in humans or in GBS.

## GBS Variants

Currently, GBS is viewed as an autoimmune condition with a diverse spectrum of presentation. The unique variants of GBS, namely AIDP, acute motor axonal neuropathy (AMAN), acute motor-sensory neuropathy (AMSAN), Miller Fisher syndrome/Bickerstaff brainstem encephalitis (MFS/BBE), and ataxic GBS/acute sensory ataxic neuropathy have differing immunological causes and will be discussed further in the present review.

## Acute Inflammatory Demyelinating Polyradiculoneuropathy (AIDP)

The most common variant of GBS in North America and Europe is AIDP (Suzuki, 2013). AIDP was also the most common variant in a study of GBS in the elderly (Peric et al., 2016). As its name implies, it is characterized by inflammatory demyelination and when it is very severe, it can also lead to secondary axonal degeneration (Griffin et al., 1996; Ho et al., 1998; Jasti et al., 2016).

### Molecular Mimicry

In a study examining the prevalence of *C. jejuni* infections in GBS cases in rural China, it was found that of those diagnosed with AIDP, 42% of their sample was seropositive for a previous *C. jejuni* infection (Ho et al., 1995). Given that γδ T cells may be underlying the response to *C. jejuni* due to their ability to recognize non-protein epitopes, Cooper et al. performed a preliminary examination of the reactivity of PBMCs isolated from GBS patients to a *C. jejuni* strain cultured from a single patient with GBS (Cooper et al., 2002). They found that there was an increase in proliferation in PBMCs from two of the GBS patients. In subsequent sural nerve biopsies from eight of the patients who had *C. jejuni* infections themselves, there was an increase in the expression of CD1b (necessary for the presentation of non-protein antigens to γδ T cells). This was in keeping with findings by Khalili-Shirazi et al. who found CD1b expression on endoneurial macrophages in two AIDP patients (Khalili-Shirazi et al., 1998). Along with CD1b expression there was also an infiltration of γδ T cells, CD8^+^ T cells, and macrophages and an increase in the expression of HLA-DR.

*C. jejuni* is not the only bacteria associated with AIDP. A preceding infection with *M. pneumoniae* has been associated with the presence of galactocerebroside antibodies (Kusunoki et al., 2001). Moreover, there is a galactocerebroside-like structure on *M. pneumoniae* which could be leading to molecular mimicry in AIDP. Definitive data that *C. jejuni* or *M. pneumoniae* has a causative role in AIDP is still however outstanding.

### Immunological Mechanism

In early studies examining the pathogenesis of GBS, it was thought that the main immunological players were macrophages and lymphocytes, since the majority of autopsy and biopsy studies found marked infiltration of macrophages in the basement membrane that appeared to be stripping myelin from axons (Prineas, 1981). The current thinking regarding the mechanism underlying the demyelinating aspect of AIDP is that complement activation is aimed at the surface of SCs (Hafer-Macko C. E. et al., 1996) that would subsequently lead to breakdown of myelin and thus recruitment of macrophages (Jasti et al., 2016). Hafer-Macko et al. have indeed shown in an autopsy study that individuals with AIDP did display C3d and membrane attack complex (MAC) deposition on the surface of their SCs (Hafer-Macko C. E. et al., 1996). This study suggested that complement was involved in the pathology of AIDP and the authors speculated that complement activation could be caused by antibody binding that leads to myelin breakdown and macrophage recruitment. As reported, these macrophages could penetrate the basal lamina of the SCs, dissociate the SCs, and then begin to strip away myelin (Griffin et al., 1996).

As T lymphocytes were also observed in autopsy and biopsy studies, Wanschitz et al. aimed to dissociate the role of complement and T cells in AIDP by using autopsy material to examine complement components and their regulators (i.e., CD59) as well as infiltrating T cells (Wanschitz et al., 2003). Like Hafer-Macko et al., the investigators found an increase in complement deposition along myelinated fibers as well as an increase in C9neo antigen (a component of MAC) in areas of demyelination. Interestingly, CD59 was upregulated where demyelination was taking place.

Within the endoneurium, there was a further correlation with the degree of demyelination and the number of CD3^+^ T cells. There is some evidence that these T cells may recognize self antigens. In one study, lymphocytes from AIDP patients and other disease controls were exposed to Protein (P) 2 protein, crude human peripheral nerve, or CNS myelin basic protein (MBP) and assayed for macrophage migration inhibitory factor (MIP) (Sheremata et al., 1975). The authors found that only AIDP patient lymphocytes showed sensitization to P2 or crude peripheral nerve. Others have also found that PBMCs from some GBS patients show reactivity to myelin protein zero (MPZ/P0) (Khalili-Shirazi et al., 1992). Further, when sera from patients with AIDP was applied to teased mouse sciatic nerves, 44% showed IgG bound to the nodes of Ranvier or the paranodes. Others have also noted that patient sera significantly contained anti-gliomedin antibodies (Devaux et al., 2012) and that anti-LM1 antibodies associate with demyelinating GBS (Kuwahara et al., 2011).

In addition to immune cells, SCs have been found to possess the necessary components to process and present antigens when cultured *in vitro* with IFN-γ (Meyer Zu Horste et al., 2010). These include proteasome subunit delta (Y), TAP2, HLA Class I, and HLA-DP, DQ, DR. Some evidence that this may occur *in vivo* was the discovery in sural nerve biopsies from AIDP patients that SCs were found to be co-localized strongly with proteasome subunit delta (Y), TAP2, HLA Class I, and HLA-DP, DQ, DR compared to control nerves.

### Discussion: Possible AIDP Mechanism(s) Including the Plausible Role of Aging

As mentioned previously, γδ T cells were identified in nerve biopsies from two of two AIDP patients and may represent a pathological population in AIDP due to their ability to recognize non-protein epitopes. While this represents a very small sample size, γδ T cell recognition of *C. jejuni* would strengthen the molecular mimicry hypothesis. This possibility is strengthened by the observation that CD1b expression, which is necessary for antigen presentation to γδ T cells, is increased in patients who had previous *C. jejuni* infections (Cooper et al., 2002). In addition to a possible involvement of γδ T cells, several groups have identified complement activation in areas with active demyelination in AIDP patients (Hafer-Macko C. E. et al., 1996; Wanschitz et al., 2003); specifically, complement products have been localized to the surface of SCs (Hafer-Macko C. E. et al., 1996). This complement activation is believed to have been triggered by antibody binding to glycoconjugates on the surface of SCs. It is plausible that this antibody-driven activation could explain the increased presence of macrophages seen in AIDP autopsy and biopsy samples (Prineas, 1981). Furthermore, given that previous studies have shown that T cells from AIDP patients have a sensitization to crude peripheral nerve and P2 protein, it stands to reason that these sensitized T cell could be initiating B cell activation and antibody production.

With respect to aging, Márquez et al. recently assessed for sex differences and age-related changes of the immune system in humans (Márquez et al., 2020). In regard to B cells, the authors found that older males display a decrease in the percentage of B cells compared to young males and older females. Further, with age, there were alterations in the B cell repertoire (Weksler and Szabo, 2000), and antibody production in response to vaccination was lessened in the elderly relative to the young (Stiasny et al., 2012). In the latter however, the responsiveness and functionality of antibodies produced did not appear to be affected by age (Stiasny et al., 2012). In keeping with this, Pritchard et al. found no change in B cell populations in GBS relative to controls (Pritchard et al., 2007). Aside from a decline in B cells, Marquez et al. also found that males appear to have accelerated age-related transcriptomic changes to their PBMCs compared to females (Márquez et al., 2020). Given that GBS affects more males than females, this could imply that the immune changes seen with aging in males may be a contributing factor to the pathogenesis of GBS via alterations in B cells. In addition, there have been some studies suggesting that γδ T cells and B cells interact (Häcker et al., 1995; Rampoldi et al., 2020). More specifically, Häcker et al. have found that when autologous B cells were used to stimulate γδ T cells in culture, it led to an increase in the percentage of Vδ1^+^ cells (Häcker et al., 1995). This is of particular interest given that Borsellino et al. noted an increased prevalence of the Vδ1 subset in GBS patients (Borsellino et al., 2000).

### Experimental Allergic Neuritis (EAN) Model

The majority of the mechanistic information gleaned about AIDP has come from work in an animal model known as EAN. EAN was first described in 1955 by Waksman and Adams (Waksman and Adams, 1955) and consists of immunizing rodents with whole myelin or specific myelin proteins, or through the adoptive transfer of T cells sensitized to the P2 myelin antigen, similar to the CNS equivalent model experimental autoimmune encephalomyelitis (EAE) (Izumo et al., 1985). As the disease progresses, the rodents experience weight loss and increasing weakness and paralysis until a peak is reached, followed by a gradual recovery phase. Support that EAN was a useful model for AIDP was provided by Asbury et al. who drew many similarities between the inflammatory infiltrate seen in autopsied AIDP tissue and that in EAN (Asbury et al., 1969). More specifically, during the clinical course of EAN, CD4^+^ T cells can be seen in the sciatic nerve in detectable numbers prior to the onset of clinical symptoms (Zhu et al., 1997). At the peak of disease, there is a steep increase in the number of CD4^+^ cells that then eventually decreases to slightly above baseline levels around the beginning of the recovery phase. CD8^+^ T cells, albeit much less than CD4^+^ T cells, can also be seen in EAN. However, they are not detected until the peak of the disease and they themselves do not peak until the initial phases of recovery.

In terms of disease resolution, it has been found that apoptosis of T cells accounts for the eventual reduction of approximately 19% of the T cell infiltrate seen at peak EAN (Zettl et al., 1994). Using the adoptive transfer model of EAN, Mausberg et al. developed a P2-sensitized T cell line expressing green fluorescent protein (GFP) so as to track the kinetics of these cells in EAN (Mausberg et al., 2018). Following transfer, the authors found that T cells enter the PNS in two distinct waves. The first wave occurs from day 1 to day 3 and is composed of endogenous T cells close to but not within the nerve environment. Around day four, there is another wave made up of both transferred and endogenous T cells found close to the nerves, however in this instance, only the transferred T cells were able to cross into the endoneurium from the epineurium.

In addition to mice, EAN has been modeled in rats. Using adoptive transfer, Hahn et al. injected T cells sensitized to P2 peptide and antibodies against galactocerebroside into naïve Lewis rats (Hahn et al., 1993). They found extensive immune infiltration and demyelination and from this concluded that when T cells enter the peripheral nerve environment, they alter the BNB. The alterations to the BNB open up the endoneurium to an attack via circulating antibodies to nerve antigens. Of note, EAN in rats also involves a very strong B cell response with an increase in antibody-producing cells prior to disease onset and which peaked at peak disease (Zhu et al., 1994).

In terms of the molecular factors underlying EAN etiology in rats, MAC (C5b-9) has been found to be deposited on the surface of SCs and myelin sheaths prior to the onset of symptoms in EAN (Stoll et al., 1991). Clusterin, a protein involved in lipid transport, membrane recycling, and regulation of injury-induced attack of the complement on membranes, has been found to reduce the severity of the disease course of EAN but did not change the initial onset of the disease (Dati et al., 2007). In addition, when the nerves were examined histologically, it was found that the clusterin-treated nerves showed significantly more remyelinated fibers compared to controls showing that its administration was more effective in the recovery phase compared to the induction phase of EAN.

In addition to prominent infiltration of T cells during the disease course of EAN, macrophages are also involved. Hartung et al. have demonstrated that when the production of eicosanoids by macrophages is inhibited, the disease course of EAN is attenuated (Hartung et al., 1988). Macrophages may also contribute to the regulation and apoptosis of T cells via the secretion of TNF-α, reactive oxygen and nitrogen species, or direct contact (Wu et al., 1995; Aliprantis et al., 1996; Kiefer et al., 2001). During recovery, macrophages may also contribute via functions seen in Wallerian and Wallerian-like degeneration (e.g., myelin debris clearance) (Kiefer et al., 2001).

Several studies have also identified a role for mast cells in the course of EAN. Brosman et al. examined the presence of mast cells over the course of EAN in Lewis rats and found that at 8 days post EAN-induction, there was a decrease in the number of mast cells present compared to control animals (Brosman et al., 1985). In addition to number of mast cells, the authors also examined the percentage of degranulated mast cells and found a significant increase from controls at 9 days post EAN-induction. The decrease in mast cell counts and the increase in the percentage of degranulated mast cells occurred prior to symptom onset (10 days post-induction). Later in the disease course (~3 weeks post-induction), there was an increase above control levels in the number of mast cells as well as the percentage of degranulated mast cells that persisted until 16 weeks post-induction; although the authors pointed out that these cells could have actually been a combination of mast cells and basophils. Interestingly, Seeldrayers et al. attempted to hinder the functioning of mast cells by giving an anti-inflammatory drug called nedocromil sodium which is known to stabilize mast cells starting at 7 days post-EAN induction (Seeldrayers et al., 1989). When treated with the drug, there was a 50% reduction in the incidence of EAN. Further, at 15 days post-induction, the average clinical score was significantly lower in the treated group (average score of 1.02) compared to the saline-treated group (average score of 1.99). Therefore, there appears to be a role for mast cells in the initiation of EAN, possibly in alterations to the BNB that allow for their penetration into the nerve environment.

#### Integrins in EAN

The role of integrins has been examined in EAN. At peak disease, it was found that myelinating SC units were abnormal in that staining revealed many broken myelin rings with uniform staining. Further, these glia lost α2 and β4 integrins unlike in GBS where only β4 integrins were absent (Previtali et al., 1998). Similar absence of α2 and β4 integrins was seen in non-myelinating SCs as well. Following peak disease, β4 integrins were still not present while α2 integrins were re-expressed on SCs again. In the aforementioned study, the authors also induced a more severe form of EAN through a higher dose of myelin and found extensive myelin degradation and axonal loss as well as the appearance of α5 expression on SCs.

In addition to these integrins, others have looked at very late antigen-4 (VLA-4 or α4/β1 integrin) and vascular cell adhesion molecule-1 (VCAM-1) in EAN (Enders et al., 1998). At the onset of clinical symptoms, VCAM-1 expression was present and increased indicating a role in disease progression. Regarding VLA-4, it was found that when VLA-4 antibodies were given in an adoptive transfer EAN model, the treatment significantly weakened the disease severity and was associated with minimal T cell or macrophage infiltration. This was also the case when VCAM-1 antibodies were given but less strongly than VLA-4. Intercellular adhesion molecule-1 (ICAM-1) has also been implicated in EAN (Stoll et al., 1993). It was found to be expressed on endothelial cells and on infiltrating macrophages at or just before the commencement of clinical symptoms. Endothelial-leukocyte adhesion molecule-1 (ELAM-1) has also been found to be increased in GBS patient sera (Hartung et al., 1994).

#### Matrix Metalloproteinases (MMPs)

Changes in MMPs have been reported in EAN. MMPs are endopeptidases that contain zinc and can be secreted by macrophages and T cells (Goetzl et al., 1996). Among many actions, MMP effector functions in the context of the immune system can include facilitating T cell migration across the basement membrane and activation of TNF-α from its membrane-bound form via cleavage. In EAN, the mRNA for a 92 kD gelatinase (MMP9) was found to peak at peak disease and then decrease through recovery (Hughes et al., 1998; Kieseier et al., 1998). Matrilysin (MMP7) also peaked at peak disease but remained elevated thereafter unlike MMP9. Macrophage metalloelastase followed a similar expression pattern as MMP7, however the maximum of macrophage metalloelastase was drastically lower than MMP7 (Hughes et al., 1998). Alterations in stromelysin-1 mRNA levels have also been reported where it began increasing with symptom onset, peaked at peak disease and then decreased sharply as recovery begins.

#### Cytokines

Not surprisingly because of the robust immune response, there are changes in cytokine levels in EAN. In an examination of mRNA levels of IFN-γ, IL-1β, IL-12, and IL-10 over the time course of EAN, Gillen et al. found that at symptom onset, there was an increase in IFN-γ, IL-1β, and IL-10 mRNA (Gillen et al., 1998). IFN-γ mRNA expression decreased in the following days but persisted at low levels for the duration of the disease. IL-10 mRNA was above control levels for most of the disease but decreased in the early recovery phase while IL-1β was at its highest at symptom onset and decreased across the disease course. Lastly, IL-12 did not show an increase until peak disease and persisted until the recovery phase. Based on these findings, the authors made several conclusions. Because IFN-γ and IL-1β mRNA expression was increased at the onset of symptoms, this could mean that the two pro-inflammatory cytokines may be playing a role in the amplification of the immune response during EAN. IL-12 is typically an inducer of IFN-γ, however in this study, it did not reach its peak mRNA expression until after IL-12, therefore more experiments need to be performed to elucidate its function in the context of EAN.

In contrast to the pro-inflammatory cytokines, IL-10 may be playing a role in disease recovery as is TGF-β1 which are upregulated by macrophages and may act as an immunosuppressant in EAN (Kiefer et al., 1996). Some direct evidence that IL-10 has an immunosuppressive effect in EAN is the reduction in symptom severity seen when IL-10 is administered at immunization or after disease onset (Bai et al., 1997). In a similar study examining the number of immune cells expressing cytokine mRNA during EAN, Zhu and colleagues corroborated the previously mentioned findings in regard to IL-1β and IL-12 (Zhu et al., 1997). For IL-10 however, they reported that the number of IL-10 mRNA-expressing cells began increasing at peak disease and increased through recovery to reach its peak at the end of the disease course. Unlike the aforementioned studies, Zhu et al. also examined IL-6, TNF-α, TNF-β, and cytolysin mRNA expression. The number of IL-6 mRNA expressing cells followed a similar pattern as IL-1β in that it peaked around the time of disease onset and gradually declined over the course of the disease. However, when examining the number of IL-6-producing cells, the peak occurred at peak disease. Similarly, the number of TNF-α mRNA expressing cells began increasing prior to symptom onset and peaked at peak disease. TNF-β expression also followed a similar trajectory as TNF-α, but with a smaller number of expressing cells. Lastly for the number of cytolysin-expressing cells, there was a peak at the onset of recovery with a slight decrease as recovery continued. An important caveat to all of these studies however is that they were examining the expression strictly in EAN and were not comparing relative to controls. This is important to note because many of these cytokines are expressed in Wallerian degeneration and may simply be an indication of that process (Kiefer et al., 2001).

IL-23p19 is another cytokine observed in EAN (Hu et al., 2006). Its mRNA first appeared prior to symptom onset, peaked at the onset of symptoms and decreased through the beginning phases of disease recovery. The authors then examined sural nerve biopsies of AIDP patients and found that the cellular source of IL-23p19 was endoneurial macrophages. Because the expression of IL-23p19 was detected prior to the infiltration of immune cells associated with symptom onset, the authors concluded that the resident macrophages are the likely cellular source of this cytokine.

While it has been found that there is an increase in the expression of IFN-γ mRNA in the initial stages of EAN, others have shown that if IFN-γ is knocked out, there is significantly worse disease following the acute stage (Zhang et al., 2012). The authors of this study were attempting to elucidate the role of Th17 cells in EAN by knocking out the potent Th1 cytokine, IFN-γ. Indeed, cultured macrophages from the null animals displayed reduced production of nitric oxide and Th1 cytokines (IL-6 and IL-12) which implied a reduced Th1 response. As such, it was surmised that the worse disease in the knockout mice was likely due to an increase in IL-17A-expressing CD4^+^ cells infiltrating the cauda equina compared to controls. Further, lymphocytes isolated from the knockout mice also had increased proliferative responses to P0 which was used to induce EAN in this study. This study highlighted a potential role for Th17 cells in EAN. To further distinguish the role of Th17 cells in EAN, Wang et al. examined cytokine expression in EAN (Wang et al., 2014). Compared to symptom onset, there was a greater proportion of both CD4^+^ IL-17A^+^ and CD4^+^ IL-22^+^ cells at peak disease. In addition, they found the expression of cytokines IL-17A and IL-22, and transcription factor RORγt to be increased at peak compared to onset. Opposite to this and in alignment with disease progression, there was a decrease in the proportion of Tregs at peak compared to onset.

#### Chemokines

Chemokines play critical role in immune cell chemotaxis. To identify if specific chemokines are involved in EAN pathogenesis, Zou et al. examined expression of macrophage inflammatory protein-1α (MIP-1α), MIP-2, and monocyte chemotactic protein-1 (MCP-1) in EAN (Zou et al., 1999). MIP-1α peaked at the peak of disease and then steadily decreased, while MCP-1 peaked just prior to symptom onset and then decreased thereafter until the beginning of the recovery phase. MIP-2 began increasing at peak disease and then peaked in the beginning of the recovery and then decreased into full recovery. In this study, the authors also gave anti-chemokine antibodies and examined the effect on EAN progression. Anti-MIP-1α delayed the onset of the disease and significantly weakened the severity. Similarly, MCP-1 antibodies also delayed disease onset and minimized the severity, but much less so than the anti-MIP-1α treatment. Lastly, MIP-2 did not have a significant effect on either the onset or the severity of EAN.

Expanding on this work, Xia et al. examined the prevalence of pro-inflammatory chemokine ligand and receptor pairs in a severe model of EAN (Xia et al., 2010). Using qPCR, the authors found significant increases in the level of CCL2/CCR2, CXCL10/CXCR3 and CCL5/CCR1, and CCR5 in EAN mice compared to controls. When looking at the cellular source, the authors found that CXCL10 co-localized with endoneurial microvessels while its receptor co-localized with CD3^+^ T cells. They also noted that CCL2 was expressed on SCs with its receptor CCR2 co-localizing with macrophages and occasionally CD3^+^ T cells. Lastly, CCL5 was expressed on axons while macrophages and CD3^+^ T cells also occasionally co-localized with the receptors, CCR1 and CCR5.

#### Discussion: Possible EAN Mechanisms and the Plausible Involvement of Aging

Finally, age may be important player in EAN. In a study examining the pattern of EAN in different ages of rats, the authors found that younger rats (i.e., 4 weeks old) had less severe disease with more frequent relapses compared to older rats (i.e., 25 weeks old) (Brosnan et al., 1988). In the older rats, their disease was acutely severe, but monophasic in disease course. When the animals were challenged a second time 44 days after the initial disease induction, the older rats showed a major effect while the younger rats had a mild reaction to the re-challenge with some instances of relapse. It is possible that the decreased responsiveness of macrophages to IL-4 with increasing age, may explain the increased severity of EAN in older animals (Brosnan et al., 1988). The authors however cautioned that due to the differences in disease patterns in EAN compared to GBS, their findings should be accepted with caution.

Differences in EAN between newborn and adult Lewis rats were also examined by Pilartz et al. (2002). They found that adoptive transfer of EAN in newborn rats led to disease induction characterized by immune infiltration and mast cell degranulation. Although this immune infiltration was present, in the newborn rats they were unable to identify any instances of demyelination possibly due to the relative size differences of myelinated fibers between adult and newborn animals.

In general, the mechanism underlying EAN is very similar to AIDP, as one would hope an animal model would be. In EAN, it appears that T cells and mast cells play a role in the opening of the BNB since sensitized T cells are able to enter into the nerve (Mausberg et al., 2018). Because adoptive transfer studies of T cells sensitized to P2 protein have shown effective transfer of EAN, it stands to reason that T cell-mediated attack on peripheral nerve proteins would underlie the model. Following the contributions of T cells (Hahn et al., 1993) and mast cells in opening the BNB (Olsson, 1967; Esposito et al., 2002), this would then allow for the infiltration of antibodies and complement products that would further contribute to the demyelination by attacking the SC surface (Stoll et al., 1991). As noted by Zhu et al., EAN in rats has been associated with a robust B cell response and increased antibody production in the early phases of the model (Zhu et al., 1994). Because it has been shown that with increasing age there is an increased presence of macrophages and mast cells within the peripheral nerve (Ceballos et al., 1999), it is plausible that these cells could contribute to the increased severity seen with age in EAN. Notably, if there is an increased presence of these cells already within the nerve then the initial attack by T cells may trigger a very robust and acute response due to the immediate proximity of these other cell types.

## Acute Motor Axonal Neuropathy (AMAN) and Acute Motor-Sensory Neuropathy (AMSAN)

AMAN and AMSAN are referred to as the axonal variants of GBS and were not properly defined until the early 1990s (Feasby, 1994). AMAN is the most common variant in Asia and South America (Kuwabara and Yuki, 2013). Interestingly, there are seasonal variations in the prevalence of AMAN; for example in China, acquisition of the disease coincides with increases in *C. jejuni* infections (Ho et al., 1995; Hafer-Macko C. et al., 1996). In AMAN, the target of the immune attack is motor fibers, while in AMSAN the target is both motor and sensory fibers (Griffin et al., 1995, 1996). AMAN has been found to have a more rapid onset compared to AIDP (Kuwabara and Yuki, 2013), and further, it is believed that AMSAN is a more severe form of AMAN (Griffin et al., 1996). Interestingly, in AMAN there is minimal demyelination and lymphocyte activity, and as such it is speculated to be an antibody- and complement-mediated attack on peripheral axons (Hafer-Macko C. et al., 1996). Patients with AMAN tend to recover rapidly, and it has been speculated that the motor deficits seen may be caused by antibody binding at the nodes of Ranvier (Ho et al., 1997). Of note, AMSAN is usually seen in older patients as compared to AMAN (Webb et al., 2015). That is, the rate of incidence of AMSAN in an elderly group of patients was twice that of a young cohort (12% vs. 6%) (Peric et al., 2016). However, in another group of AMAN patients, it was found that 58% of those presenting with antibodies to nodal adhesion molecules were over the age of 60 (Devaux et al., 2012).

### Immunoglobulins

A current theory as to the cause of AMAN and AMSAN is molecular mimicry. The strong association between *C. jejuni* infections and AMAN has been shown to be caused by the presence of Gal(β1-3)GalNAc in the lipopolysaccharides of some strains of *C. jejuni*. This particular epitope is also found in GM1 gangliosides that are localized at nodes of Ranvier (Hafer-Macko C. et al., 1996). In conjunction with this, those with AMAN have been found to have IgG antibodies against GM1, although not in every case. To demonstrate that the antibodies and complement were involved in the pathogenesis of AMAN, Hafer-Macko et al. performed post-mortem examinations of AMAN patients and found the presence of IgG antibodies and the complement product C3d present at the nodes of Ranvier, specifically bound to the axolemma (Hafer-Macko C. et al., 1996). They postulated that the activation of complement leads to the recruitment of macrophages that target axons whereupon they enter the periaxonal space, displace axons, and separate them from their myelin sheaths. In some cases, this led to axonal degeneration. Findings of macrophages entering the periaxonal space and displacing axons has been found by other groups as well and likely explains the deficits in nerve conduction studies see in AMAN patients (Griffin et al., 1995, 1996).

In keeping with these findings and similar to that seen in AIDP, when AMAN patient serum was bathed onto teased mouse sciatic nerves, 42% showed IgG deposition at nodes of Ranvier and paranodal regions with a significant percentage of patient sera containing antibodies directed against NF186 (Devaux et al., 2012). GM1 gangliosides have also been found to increase TrkA autophosphorylation induced by nerve growth factor (Tanaka et al., 2007). When IgG from the sera of four AMAN patients was added to a PC12 sympathetic nerve cell line pre-incubated with GM1, neurite outgrowth was arrested. The same effect was seen in cells treated with nerve growth factor prior to the addition of IgG. Further, when patient IgG was depleted of anti-GM1 antibodies, the effect on neurite outgrowth was no longer seen. Other antibodies associated with AMAN include GD1a, GM1b, and GalNAc-GD1a (Willison and Yuki, 2002; Suzuki, 2013) whereas they are minimally present in AIDP (Ho et al., 1999).

### Role of Gangliosides in AMAN, AMSAN, and Animal Models

Greenshields et al. went further to show that the antibody-binding domain of GM1 gangliosides can be shielded by the presence of other gangliosides such as GD1a in ganglioside complexes in cell membranes (Greenshields et al., 2009). In their study, they found that the ability of antibodies to bind to GM1 and other ganglioside complexes was inhibited to varying degrees depending on the antibody and the second ganglioside in the complex. The masking effect prevented binding and complement activation and this effect was reversed when treated with enzymes that exposed the domain as evidenced by increased MAC and C3 deposits.

Because there have been associations with the gangliosides, GM1 and GD1a, in AMAN, Susuki et al. examined the nodes of Ranvier of mice lacking these gangliosides and found many structural changes in the nodal region including changes in ion channel localization (Susuki et al., 2007a). For instance, potassium channels were located in the paranodes rather than juxtaparanode and there were broader clusters of sodium channels. Other structural deficits were also noted such as unattached paranodal loops and weakened staining of Caspr and NF155 compared to control mice. Interestingly, the authors found that as the knockout mice aged, there were increased abnormalities in nodal structure implying that GM1 and GD1a must be involved in the maintenance of the nodal region with age. A similar study examining mice with an ablation for GalNAc-transferase specifically from SCs, showed that although development was normal, the animals displayed reduced myelin and an increase in axon degeneration with increasing age (Yao et al., 2014). Thus, antibody-mediated insults on peripheral nerve gangliosides may carry with them greater implication for the severity of GBS because of their importance in nerve maintenance with age.

Recently, a transgenic mouse model has been created for AMAN by knocking out complex gangliosides everywhere but on neurons (McGonigal et al., 2016); this allows for the specific targeting of antibodies against gangliosides. When given anti-GM1 antibodies and normal human serum, the transgenic mice developed a phenotype similar to that seen in AMAN. Interestingly, when anti-C1q antibodies were given to block the classical complement cascade, there was no detectable complement activation products which coincided with increased axonal integrity compared to untreated mice. However, this did not impact immune cell recruitment between treated and untreated mice as there were no significant differences in the number of neutrophils or CD11b^+^ leukocytes. This transgenic mouse line has also been used to demonstrate that neurons are capable of greatly reducing the levels of circulating anti-ganglioside antibodies via endocytosis (Cunningham et al., 2016). Specifically, when given injections of two different monoclonal antibodies against GM1 (one that is inhibited from binding due to shielding by neighboring gangliosides, and one with a strong binding ability regardless of neighboring gangliosides), only the strongly binding antibody was reduced from circulation indicating that the antibody must be bound to the membrane in order to be endocytosed by the cell. It was further found that this occurs most prominently at the presynaptic terminal of the neuromuscular junction (NMJ). Altogether, these findings could account for the variation in antibody presence in GBS patient sera.

In animal models of AMAN, IgG antibodies have been found to bind to both the nodal and paranodal regions of the axon (Shahrizaila and Yuki, 2013). This led to alterations in sodium channel presence in addition to a detachment of myelin. Others have shown that anti-GM1 antibodies are able to disrupt sodium channels at nodes of Ranvier through the activation of complement, and as recovery progresses, complement depositions decrease and sodium channels normalize (Susuki et al., 2007b). Furthermore, when inhibitors of the complement are given, sodium channels are unaltered after anti-GM1 sensitization (Phongsisay et al., 2008).

Yuki et al. have developed a model of the axonal variants of GBS (AMAN and AMSAM) in rabbits through the immunization of a mixture of bovine brain gangliosides or GM1, Freund's adjuvant, and keyhole limpet hemocyanin (KLH) (Yuki et al., 2001). The rabbits experienced axonal degeneration with no demyelination or lymphocytic infiltration similar to that seen in axonal GBS. In order to determine the role of KLH in the induction of this model, Caporale et al. immunized New Zealand white rabbits with lipopolysaccharide from the isolated *C. jejuni* of GBS patients with GM1 and GD1a epitope expression and Freund's adjuvant with or without KLH (Caporale et al., 2006b). They found that in the KLH group, 6 out of 7 rabbits developed disease while only 1 out of 11 from the non-KLH group developed disease. The authors also noted that in both groups, the rabbits developed high volumes of antibodies against lipopolysaccharide and GM1 and low volumes of antibodies against GD1b and GD1a. While they were unable to determine the exact function of KLH in this model, they speculated that it may play a role in T cell recruitment and activation.

In another model of AMAN, Yuki et al. have demonstrated that immunization of rabbits with lipo-oligosaccharides derived from *C. jejuni* led to the development of anti-GM1 antibodies and clinical symptoms similar to those seen in human axonal GBS (Yuki et al., 2004). Furthermore, in their study, the authors immunized mice lacking the enzymes necessary to develop complex gangliosides with lipo-oligosaccharides derived from *C. jejuni* and found that the mice developed monoclonal antibodies against GM1. The specificity and purity of the lipo-oligosaccharides is critical for the proper induction of the model (Notturno et al., 2010). When two lipo-oligosaccharides were derived from the same strain of *C. jejuni*, only the purer of the two induced the symptoms. Both groups developed antibodies against lipo-oligosaccharides, however significantly higher levels were seen in the disease-induced group. Both cohorts also developed IgM and IgG anti-GM1, anti-GD1a, and anti-GD1b, however no significant differences were seen between the two groups although there were significant differences in the neuropathy developing group which possessed high levels of monospecific and high-affinity anti-GM1 antibodies not seen in the other rabbits. In addition, the sera of rabbits which developed the neuropathy were able to activate complement as assessed by higher deposition of C3b/c.

Aside from anti-GM1 antibodies, anti-GD1a antibodies have also been associated with AMAN. In a mouse model, it has been found that there is a preference for anti-GD1a antibody binding in more distal nodes of Ranvier on motor nerve bundles due to the added protection of the BNB at more proximal sites (McGonigal et al., 2010). When antibody binding occurs at these sites, it leads to complement activation and MAC pore deposition which in turn causes calpain activation. This results in a loss of Nav1.6 channels and thus loss of action potentials. However, when axonal structures are preserved through the inhibition of calpains, there is still a loss of action potentials indicating that the deposition of MAC pores are causing ionic imbalances along the axon and thus preventing signal condition. Aside from complement, there is also immune cell recruitment in animal models of axonal GBS. Finally, in a study where anti-GD1b antibodies and normal human serum were used to induce neuropathy, it was found that neutrophils and macrophages were not recruited while perisynaptic SCs functioned as the main phagocyte to clear debris in distal nerve injuries induced by antibody-mediated attacks on gangliosides (Cunningham et al., 2020).

### Discussion: Mechanisms in AMAN, AMSAN, and Animal Models and Implications of Aging

Gangliosides are important for nerve health with age and an attack on these structures in older subjects may lead to even worse outcomes. This suggestion is in keeping with the incidence rates of AMAN and AMSAN with age. As noted above, AMSAN is associated with older patients relative to AMAN (Webb et al., 2015), however others have shown that 58% of AMAN patients with detectable antibodies against nodal adhesion molecules were over 60 years old (Devaux et al., 2012). Particularly in the axonal variants, it appears that antibodies against gangliosides and ganglioside complexes located at the nodes of Ranvier are critical to disease development. This leads to complement activation and further recruitment of macrophages which can further displace myelin sheaths and cause secondary axonal degeneration (Hafer-Macko C. et al., 1996). Similar to AIDP, with increasing age, there is an increase in the presence of macrophages within the peripheral nerve (Ceballos et al., 1999) which may lead to increased disease severity in the elderly in concert with decreased regenerative abilities with age.

## Anti-Gq1B Antibody Syndrome: Miller Fisher Syndrome/ Bickerstaff Brainstem Encephalitis (Mfs/Bbe)

Miller Fisher Syndrome (MFS) was first described in 1956 by Miller Fisher (Fisher, 1956). It is classically characterized by ataxia, ophthalmoplegia, and areflexia. Recently, it has been found that MFS is itself part of a spectrum that includes Bickerstaff brainstem encephalitis (BBE) (Shahrizaila and Yuki, 2013). Symptoms of BBE include ataxia, ophthalmoplegia, and differing from MFS, impairments in consciousness or hyperreflexia. What led to this discovery was the common presence of anti-GQ1b IgG antibodies in both conditions. Other anti-ganglioside antibodies associated with this spectrum include GD3 and GT1a (van Doorn et al., 2008). Similar to AMAN, there is a strong association with preceding infections (e.g., *C. jejuni*) and when lipo-oligosaccharides were isolated and cultured from patients who had a preceding *C. jejuni* infection, it was found that anti-GQ1b antibodies bound to them (Koga et al., 2005; Shahrizaila and Yuki, 2013). In the first paper to describe IgG anti-GQ1b antibodies in MFS, the authors explained that it could be a new diagnostic marker for the variant (Chiba et al., 1992) since antibodies against GQ1b have been shown to be localized to the human oculomotor, trochlear, abducens, glossopharyngeal, and vagal nerves, as well as muscle spindles which may contribute to the symptomology (Chiba et al., 1993; Kusunoki et al., 1999; Shahrizaila and Yuki, 2013). In BBE, due to the CNS involvement (i.e., altered consciousness), it is likely that the reticular formation is implicated as well (Shahrizaila and Yuki, 2013). However, others have looked at the presence of antibodies against anti-ganglioside complexes in MFS and found that some patients have serum antibodies to GQ1b-GM1, GT1a/GM1, GQ1b/GD1a, and/or GQ1b/GT1b complexes (Kaida et al., 2006) and thus it may be best to assay for a spectrum of antibodies for diagnostic purposes.

The NMJ is a vulnerable area for anti-ganglioside antibody attack due to its high concentration of gangliosides and its location outside of the BNB. Anti-GQ1b antibodies from MFS patients have been found to decrease neurotransmitter release from the presynaptic terminals (Buchwald et al., 1995). In an *ex vivo* model, anti-GQ1b antibodies have also been shown to activate complement and therefore the degeneration of the motor nerve terminals (O'Hanlon et al., 2001). In a mouse model of MFS, when given an experimental complement inhibitor, there was a lack of membrane attack complex formation on perisynaptic SCs and preserved axonal integrity (Halstead et al., 2005). This was also seen in another model when anti-GQ1b/GD3 IgM antibodies followed by normal human serum were given to wild-type mice or when anti-C1q antibodies were given to block the classical complement cascade. There was reduced activation of the complement system and deposition of complement products as well as less immune cell presence and injury to the axons (Cunningham et al., 2016). Inhibitors of complement further downstream (i.e., C5 inhibitor rEV576) were also able to protect from complement-mediated degeneration of NMJs and perisynaptic SC injury (Halstead et al., 2008a). Furthermore, as seen in the human clinical trials mentioned earlier, eculizumab (humanized monoclonal antibody against C5) was also able to protect the NMJ in the MFS mouse model (Halstead et al., 2008b).

Because complement activation leads to MAC formation, there is an influx of calcium associated with the activation of calpains within the neuron leading to degeneration of the NMJ. When given calpain inhibitors or when calcium is depleted, there is a lack of degeneration of the NMJ in this MFS model (O'Hanlon et al., 2003). Altogether, because there are some overlap of MFS with other forms of GBS such as AMAN and AMSAN in terms of types of antibodies deposited, therapies targeting such antibodies or other overlapping factors would benefit more than one disease.

### Acute Ataxic Neuropathy Without Ophthalmoplegia: Ataxic Gbs/Acute Sensory Ataxic Neuropathy

Another subgroup within the anti-GQ1b antibody syndrome GBS variant is acute ataxic neuropathy without ophthalmoplegia (Ito et al., 2011; Shahrizaila and Yuki, 2013). This subgroup is a clinical spectrum comprised of ataxic GBS and acute sensory ataxic neuropathy (Ito et al., 2011). Acute sensory ataxic neuropathy patients present with sensory ataxia without ophthalmoplegia and have a high prevalence of recent *C. jejuni* infections (Notturno et al., 2008). In addition, these patients have antibodies to GD1b and GQ1b. GD1b has been found on primary sensory neurons and in the paranodal regions of both motor and sensory nerves (Kusunoki et al., 1993), and therefore antibodies against GD1b could be targeting these sensory nerves (Pan et al., 2001). In one study, it was found that when another anti-ganglioside along with GD1b was found in the sera of GBS patients, the patients were less likely to have ataxia as a symptom (Kaida et al., 2008). From this, the authors suggested that very specific IgG antibodies against GD1b could be involved in the ataxia in GBS. In addition to having antibodies against GD1b and GQ1b as in acute sensory ataxic neuropathy, ataxic GBS involves acute ataxia with a negative Romberg sign and no ophthalmoplegia (Richter, 1962). Relative to MFS, patients in this spectrum more often have antibodies against GD1b and less often against GQ1b (Ito et al., 2011).

### Discussion: Mechanisms in Anti-GQ1b Antibody Syndrome

As mentioned above, antibodies against GQ1b are a hallmark feature of this variant and furthermore, anti-GD1b antibodies appear to be critical for the development of the anti-GQ1b antibody syndrome subtype acute ataxic neuropathy without ophthalmoplegia. GQ1b gangliosides and complexes containing it appear to localize clearly to a variety of nerves (Chiba et al., 1993; Kusunoki et al., 1999; Shahrizaila and Yuki, 2013) and the NMJ (O'Hanlon et al., 2001) that would lead to the three main symptoms ataxia, ophthalmoplegia, and areflexia. Similar to other GBS variants, antibody and complement-mediated attacks appear to be critical to the development of this variant. Interestingly, eculizumab and other complement inhibitors showed promising results in mouse models of MFS.

## Conclusion

As has been discussed above, the pathology of GBS can be characterized by abhorrent immune cell functioning wherein the host becomes the victim of its own immune response. Broadly, it appears that the attack against peripheral nerves and specific gangliosides is the product of interactions between T cells, B cells, complement products, and macrophages. In addition, the immune system and the ability of the PNS to regenerate changes over the course of the lifespan. Altogether, the combined impact of an aging immune system and a poor response to a prior infection may push the body into an altered and self-attacking state contributing to the increased frequency and severity of GBS with age.

## Author Contributions

KH and SO wrote the manuscript. All authors contributed to the article and approved the submitted version.

## Conflict of Interest

The authors declare that the research was conducted in the absence of any commercial or financial relationships that could be construed as a potential conflict of interest.
